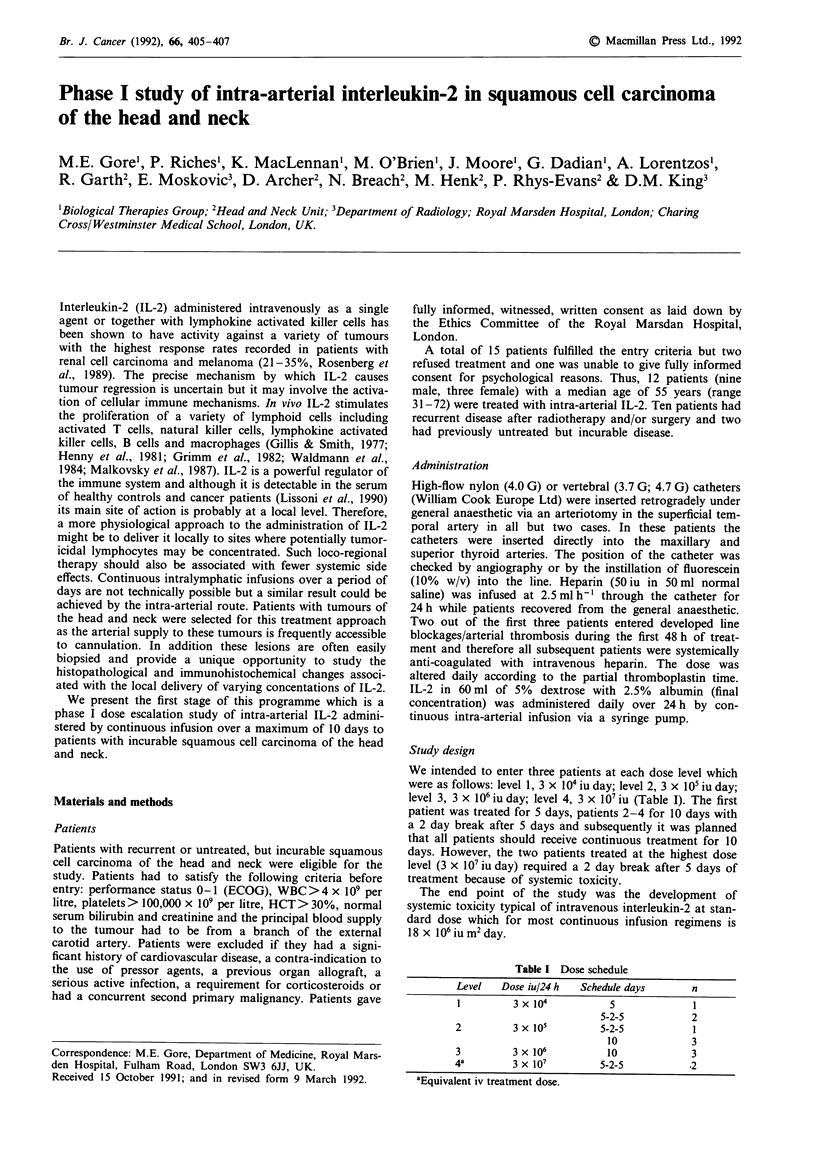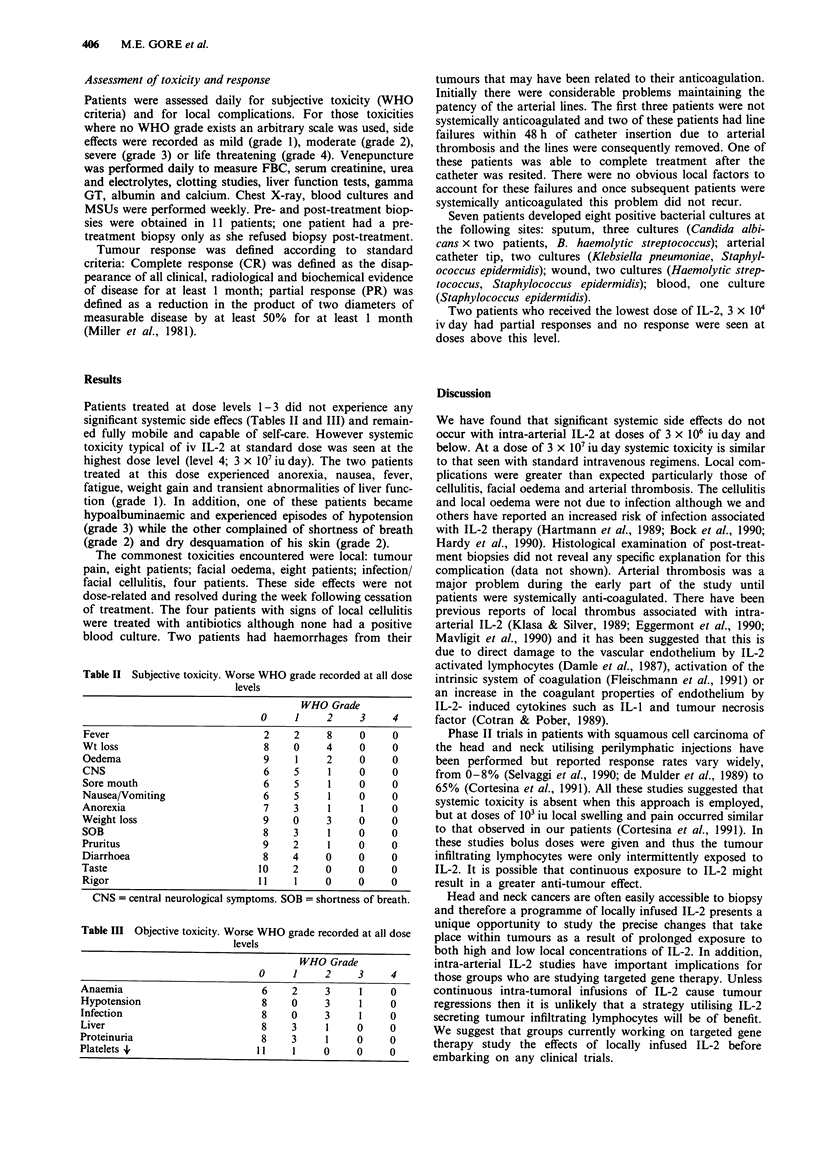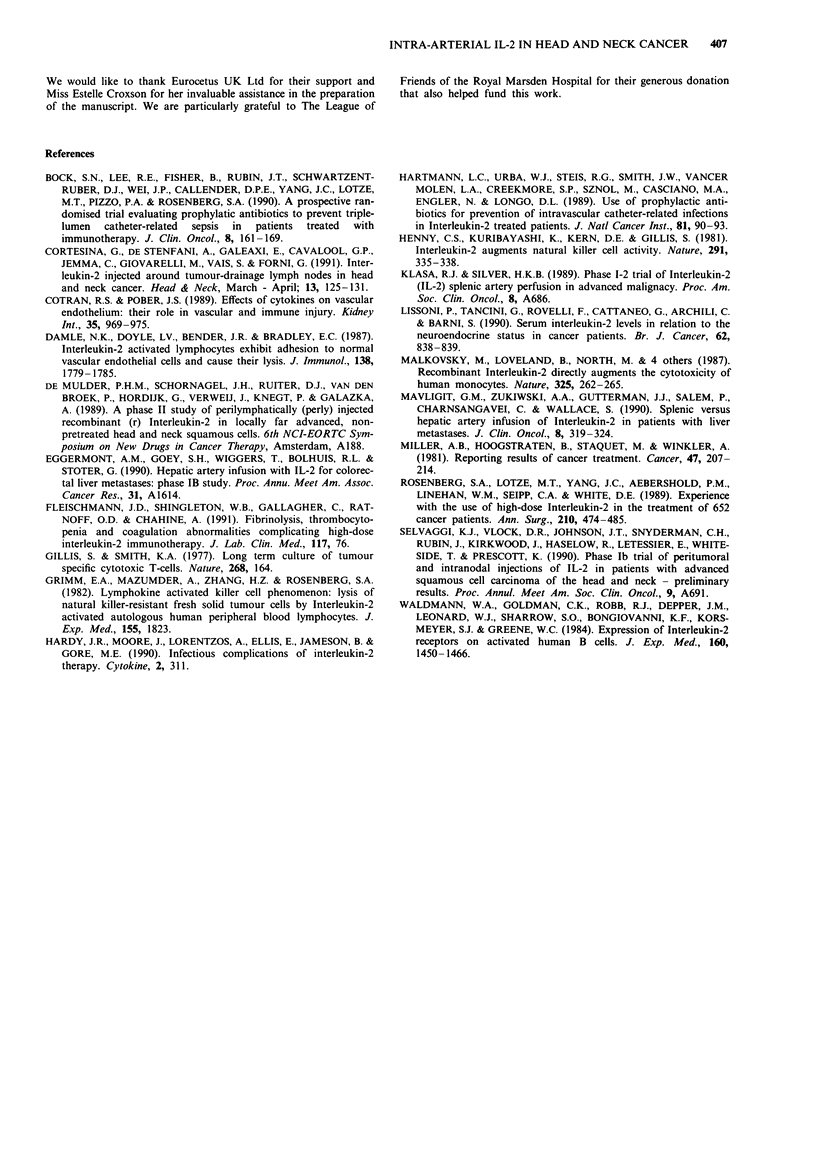# Phase I study of intra-arterial interleukin-2 in squamous cell carcinoma of the head and neck.

**DOI:** 10.1038/bjc.1992.278

**Published:** 1992-08

**Authors:** M. E. Gore, P. Riches, K. MacLennan, M. O'Brien, J. Moore, G. Dadian, A. Lorentzos, R. Garth, E. Moskovic, D. Archer

**Affiliations:** Biological Therapies Group, Royal Marsden Hospital, London, UK.


Br. J. Cancer (1992), 66, 405-407 © Macmillan Press Ltd., 1992

### Phase I study of intra-arterial interleukin-2 in squamous cell carcinoma of the head and neck

M.E. Gore[1], P. Riches[1], K. MacLennan[1], M. O'Brien[1], J. Moore[1], G. Dadian[1], A. Lorentzos[1], R. Garth[2], E. Moskovic[3], D. Archer[2], N. Breach[2], M. Henk[2], P. Rhys-Evans[2] & D.M. King[3]

[1]*Biological Therapies Group;* [2]*Head and Neck Unit;* [3]*Department of Radiology; Royal Marsden Hospital, London; Charing Cross/Westminster Medical School, London, UK.*

Interleukin-2 (IL-2) administered intravenously as a single agent or together with lymphokine activated killer cells has been shown to have activity against a variety of tumours with the highest response rates recorded in patients with renal cell carcinoma and melanoma (21–35%, Rosenberg *et al.*, 1989). The precise mechanism by which IL-2 causes tumour regression is uncertain but it may involve the activation of cellular immune mechanisms. *In vivo* IL-2 stimulates the proliferation of a variety of lymphoid cells including activated T cells, natural killer cells, lymphokine activated killer cells, B cells and macrophages (Gillis & Smith, 1977; Henny *et al.*, 1981; Grimm *et al.*, 1982; Waldmann *et al.*, 1984; Malkovsky *et al.*, 1987). IL-2 is a powerful regulator of the immune system and although it is detectable in the serum of healthy controls and cancer patients (Lissoni *et al.*, 1990) its main site of action is probably at a local level. Therefore, a more physiological approach to the administration of IL-2 might be to deliver it locally to sites where potentially tumoricidal lymphocytes may be concentrated. Such loco-regional therapy should also be associated with fewer systemic side effects. Continuous intralymphatic infusions over a period of days are not technically possible but a similar result could be achieved by the intra-arterial route. Patients with tumours of the head and neck were selected for this treatment approach as the arterial supply to these tumours is frequently accessible to cannulation. In addition these lesions are often easily biopsied and provide a unique opportunity to study the histopathological and immunohistochemical changes associated with the local delivery of varying concentations of IL-2.

We present the first stage of this programme which is a phase I dose escalation study of intra-arterial IL-2 administered by continuous infusion over a maximum of 10 days to patients with incurable squamous cell carcinoma of the head and neck.

### Materials and methods

### *Patients*

Patients with recurrent or untreated, but incurable squamous cell carcinoma of the head and neck were eligible for the study. Patients had to satisfy the following criteria before entry: performance status 0–1 (ECOG), WBC $>4 \times 10^9$ per litre, platelets $>100,000 \times 10^9$ per litre, HCT $>30\%$, normal serum bilirubin and creatinine and the principal blood supply to the tumour had to be from a branch of the external carotid artery. Patients were excluded if they had a significant history of cardiovascular disease, a contra-indication to the use of pressor agents, a previous organ allograft, a serious active infection, a requirement for corticosteroids or had a concurrent second primary malignancy. Patients gave fully informed, witnessed, written consent as laid down by the Ethics Committee of the Royal Marsdan Hospital, London.

A total of 15 patients fulfilled the entry criteria but two refused treatment and one was unable to give fully informed consent for psychological reasons. Thus, 12 patients (nine male, three female) with a median age of 55 years (range 31–72) were treated with intra-arterial IL-2. Ten patients had recurrent disease after radiotherapy and/or surgery and two had previously untreated but incurable disease.

### *Administration*

High-flow nylon (4.0 G) or vertebral (3.7 G; 4.7 G) catheters (William Cook Europe Ltd) were inserted retrogradely under general anaesthetic via an arteriotomy in the superficial temporal artery in all but two cases. In these patients the catheters were inserted directly into the maxillary and superior thyroid arteries. The position of the catheter was checked by angiography or by the instillation of fluorescein (10% w/v) into the line. Heparin (50 iu in 50 ml normal saline) was infused at $2.5 \text{ ml h}^{-1}$ through the catheter for 24 h while patients recovered from the general anaesthetic. Two out of the first three patients entered developed line blockages/arterial thrombosis during the first 48 h of treatment and therefore all subsequent patients were systemically anti-coagulated with intravenous heparin. The dose was altered daily according to the partial thromboplastin time. IL-2 in 60 ml of 5% dextrose with 2.5% albumin (final concentration) was administered daily over 24 h by continuous intra-arterial infusion via a syringe pump.

### *Study design*

We intended to enter three patients at each dose level which were as follows: level 1, $3 \times 10^4$ iu day; level 2, $3 \times 10^5$ iu day; level 3, $3 \times 10^6$ iu day; level 4, $3 \times 10^7$ iu (Table I). The first patient was treated for 5 days, patients 2–4 for 10 days with a 2 day break after 5 days and subsequently it was planned that all patients should receive continuous treatment for 10 days. However, the two patients treated at the highest dose level ($3 \times 10^7$ iu day) required a 2 day break after 5 days of treatment because of systemic toxicity.

The end point of the study was the development of systemic toxicity typical of intravenous interleukin-2 at standard dose which for most continuous infusion regimens is $18 \times 10^6$ iu m$^2$ day.

**Table I** Dose schedule

| *Level* | *Dose iu/24 h* | *Schedule days* | *n* |
|---|---|---|---|
| 1 | $3 \times 10^4$ | 5 | 1 |
| | | 5-2-5 | 2 |
| 2 | $3 \times 10^5$ | 5-2-5 | 1 |
| | | 10 | 3 |
| 3 | $3 \times 10^6$ | 10 | 3 |
| 4[a] | $3 \times 10^7$ | 5-2-5 | ·2 |

[a]Equivalent iv treatment dose.

Correspondence: M.E. Gore, Department of Medicine, Royal Marsden Hospital, Fulham Road, London SW3 6JJ, UK.
Received 15 October 1991; and in revised form 9 March 1992.

*Assessment of toxicity and response*

Patients were assessed daily for subjective toxicity (WHO criteria) and for local complications. For those toxicities where no WHO grade exists an arbitrary scale was used, side effects were recorded as mild (grade 1), moderate (grade 2), severe (grade 3) or life threatening (grade 4). Venepuncture was performed daily to measure FBC, serum creatinine, urea and electrolytes, clotting studies, liver function tests, gamma GT, albumin and calcium. Chest X-ray, blood cultures and MSUs were performed weekly. Pre- and post-treatment biopsies were obtained in 11 patients; one patient had a pre-treatment biopsy only as she refused biopsy post-treatment.

Tumour response was defined according to standard criteria: Complete response (CR) was defined as the disappearance of all clinical, radiological and biochemical evidence of disease for at least 1 month; partial response (PR) was defined as a reduction in the product of two diameters of measurable disease by at least 50% for at least 1 month (Miller *et al.*, 1981).

### Results

Patients treated at dose levels 1–3 did not experience any significant systemic side effecs (Tables II and III) and remained fully mobile and capable of self-care. However systemic toxicity typical of iv IL-2 at standard dose was seen at the highest dose level (level 4; $3 \times 10^7$ iu day). The two patients treated at this dose experienced anorexia, nausea, fever, fatigue, weight gain and transient abnormalities of liver function (grade 1). In addition, one of these patients became hypoalbuminaemic and experienced episodes of hypotension (grade 3) while the other complained of shortness of breath (grade 2) and dry desquamation of his skin (grade 2).

The commonest toxicities encountered were local: tumour pain, eight patients; facial oedema, eight patients; infection/facial cellulitis, four patients. These side effects were not dose-related and resolved during the week following cessation of treatment. The four patients with signs of local cellulitis were treated with antibiotics although none had a positive blood culture. Two patients had haemorrhages from their tumours that may have been related to their anticoagulation. Initially there were considerable problems maintaining the patency of the arterial lines. The first three patients were not systemically anticoagulated and two of these patients had line failures within 48 h of catheter insertion due to arterial thrombosis and the lines were consequently removed. One of these patients was able to complete treatment after the catheter was resited. There were no obvious local factors to account for these failures and once subsequent patients were systemically anticoagulated this problem did not recur.

Seven patients developed eight positive bacterial cultures at the following sites: sputum, three cultures (*Candida albicans* × two patients, *B. haemolytic streptococcus*); arterial catheter tip, two cultures (*Klebsiella pneumoniae*, *Staphylococcus epidermidis*); wound, two cultures (*Haemolytic streptococcus*, *Staphylococcus epidermidis*); blood, one culture (*Staphylococcus epidermidis*).

Two patients who received the lowest dose of IL-2, $3 \times 10^4$ iv day had partial responses and no response were seen at doses above this level.

**Table II** Subjective toxicity. Worse WHO grade recorded at all dose levels

| | *WHO Grade* | | | | |
|---|---|---|---|---|---|
| | *0* | *1* | *2* | *3* | *4* |
| Fever | 2 | 2 | 8 | 0 | 0 |
| Wt loss | 8 | 0 | 4 | 0 | 0 |
| Oedema | 9 | 1 | 2 | 0 | 0 |
| CNS | 6 | 5 | 1 | 0 | 0 |
| Sore mouth | 6 | 5 | 1 | 0 | 0 |
| Nausea/Vomiting | 6 | 5 | 1 | 0 | 0 |
| Anorexia | 7 | 3 | 1 | 1 | 0 |
| Weight loss | 9 | 0 | 3 | 0 | 0 |
| SOB | 8 | 3 | 1 | 0 | 0 |
| Pruritus | 9 | 2 | 1 | 0 | 0 |
| Diarrhoea | 8 | 4 | 0 | 0 | 0 |
| Taste | 10 | 2 | 0 | 0 | 0 |
| Rigor | 11 | 1 | 0 | 0 | 0 |

CNS = central neurological symptoms. SOB = shortness of breath.

**Table III** Objective toxicity. Worse WHO grade recorded at all dose levels

| | *WHO Grade* | | | | |
|---|---|---|---|---|---|
| | *0* | *1* | *2* | *3* | *4* |
| Anaemia | 6 | 2 | 3 | 1 | 0 |
| Hypotension | 8 | 0 | 3 | 1 | 0 |
| Infection | 8 | 0 | 3 | 1 | 0 |
| Liver | 8 | 3 | 1 | 0 | 0 |
| Proteinuria | 8 | 3 | 1 | 0 | 0 |
| Platelets ↓ | 11 | 1 | 0 | 0 | 0 |

### Discussion

We have found that significant systemic side effects do not occur with intra-arterial IL-2 at doses of $3 \times 10^6$ iu day and below. At a dose of $3 \times 10^7$ iu day systemic toxicity is similar to that seen with standard intravenous regimens. Local complications were greater than expected particularly those of cellulitis, facial oedema and arterial thrombosis. The cellulitis and local oedema were not due to infection although we and others have reported an increased risk of infection associated with IL-2 therapy (Hartmann *et al.*, 1989; Bock *et al.*, 1990; Hardy *et al.*, 1990). Histological examination of post-treatment biopsies did not reveal any specific explanation for this complication (data not shown). Arterial thrombosis was a major problem during the early part of the study until patients were systemically anti-coagulated. There have been previous reports of local thrombus associated with intra-arterial IL-2 (Klasa & Silver, 1989; Eggermont *et al.*, 1990; Mavligit *et al.*, 1990) and it has been suggested that this is due to direct damage to the vascular endothelium by IL-2 activated lymphocytes (Damle *et al.*, 1987), activation of the intrinsic system of coagulation (Fleischmann *et al.*, 1991) or an increase in the coagulant properties of endothelium by IL-2- induced cytokines such as IL-1 and tumour necrosis factor (Cotran & Pober, 1989).

Phase II trials in patients with squamous cell carcinoma of the head and neck utilising perilymphatic injections have been performed but reported response rates vary widely, from 0–8% (Selvaggi *et al.*, 1990; de Mulder *et al.*, 1989) to 65% (Cortesina *et al.*, 1991). All these studies suggested that systemic toxicity is absent when this approach is employed, but at doses of $10^3$ iu local swelling and pain occurred similar to that observed in our patients (Cortesina *et al.*, 1991). In these studies bolus doses were given and thus the tumour infiltrating lymphocytes were only intermittently exposed to IL-2. It is possible that continuous exposure to IL-2 might result in a greater anti-tumour effect.

Head and neck cancers are often easily accessible to biopsy and therefore a programme of locally infused IL-2 presents a unique opportunity to study the precise changes that take place within tumours as a result of prolonged exposure to both high and low local concentrations of IL-2. In addition, intra-arterial IL-2 studies have important implications for those groups who are studying targeted gene therapy. Unless continuous intra-tumoral infusions of IL-2 cause tumour regressions then it is unlikely that a strategy utilising IL-2 secreting tumour infiltrating lymphocytes will be of benefit. We suggest that groups currently working on targeted gene therapy study the effects of locally infused IL-2 before embarking on any clinical trials.

We would like to thank Eurocetus UK Ltd for their support and Miss Estelle Croxson for her invaluable assistance in the preparation of the manuscript. We are particularly grateful to The League of Friends of the Royal Marsden Hospital for their generous donation that also helped fund this work.

### References

BOCK, S.N., LEE, R.E., FISHER, B., RUBIN, J.T., SCHWARTZENTRUBER, D.J., WEI, J.P., CALLENDER, D.P.E., YANG, J.C., LOTZE, M.T., PIZZO, P.A. & ROSENBERG, S.A. (1990). A prospective randomised trial evaluating prophylatic antibiotics to prevent triple-lumen catheter-related sepsis in patients treated with immunotherapy. *J. Clin. Oncol.*, **8**, 161–169.

CORTESINA, G., DE STENFANI, A., GALEAXI, E., CAVALOOL, G.P., JEMMA, C., GIOVARELLI, M., VAIS, S. & FORNI, G. (1991). Interleukin-2 injected around tumour-drainage lymph nodes in head and neck cancer. *Head & Neck*, March - April; **13**, 125–131.

COTRAN, R.S. & POBER, J.S. (1989). Effects of cytokines on vascular endothelium: their role in vascular and immune injury. *Kidney Int.*, **35**, 969–975.

DAMLE, N.K., DOYLE, LV., BENDER, J.R. & BRADLEY, E.C. (1987). Interleukin-2 activated lymphocytes exhibit adhesion to normal vascular endothelial cells and cause their lysis. *J. Immunol.*, **138**, 1779–1785.

DE MULDER, P.H.M., SCHORNAGEL, J.H., RUITER, D.J., VAN DEN BROEK, P., HORDIJK, G., VERWEIJ, J., KNEGT, P. & GALAZKA, A. (1989). A phase II study of perilymphatically (perly) injected recombinant (r) Interleukin-2 in locally far advanced, non-pretreated head and neck squamous cells. *6th NCI-EORTC Symposium on New Drugs in Cancer Therapy*, Amsterdam, A188.

EGGERMONT, A.M., GOEY, S.H., WIGGERS, T., BOLHUIS, R.L. & STOTER, G. (1990). Hepatic artery infusion with IL-2 for colorectal liver metastases: phase IB study. *Proc. Annu. Meet Am. Assoc. Cancer Res.*, **31**, A1614.

FLEISCHMANN, J.D., SHINGLETON, W.B., GALLAGHER, C., RATNOFF, O.D. & CHAHINE, A. (1991). Fibrinolysis, thrombocytopenia and coagulation abnormalities complicating high-dose interleukin-2 immunotherapy. *J. Lab. Clin. Med.*, **117**, 76.

GILLIS, S. & SMITH, K.A. (1977). Long term culture of tumour specific cytotoxic T-cells. *Nature*, **268**, 164.

GRIMM, E.A., MAZUMDER, A., ZHANG, H.Z. & ROSENBERG, S.A. (1982). Lymphokine activated killer cell phenomenon: lysis of natural killer-resistant fresh solid tumour cells by Interleukin-2 activated autologous human peripheral blood lymphocytes. *J. Exp. Med.*, **155**, 1823.

HARDY, J.R., MOORE, J., LORENTZOS, A., ELLIS, E., JAMESON, B. & GORE, M.E. (1990). Infectious complications of interleukin-2 therapy. *Cytokine*, **2**, 311.

HARTMANN, L.C., URBA, W.J., STEIS, R.G., SMITH, J.W., VANCER MOLEN, L.A., CREEKMORE, S.P., SZNOL, M., CASCIANO, M.A., ENGLER, N. & LONGO, D.L. (1989). Use of prophylactic antibiotics for prevention of intravascular catheter-related infections in Interleukin-2 treated patients. *J. Natl Cancer Inst.*, **81**, 90–93.

HENNY, C.S., KURIBAYASHI, K., KERN, D.E. & GILLIS, S. (1981). Interleukin-2 augments natural killer cell activity. *Nature*, **291**, 335–338.

KLASA, R.J. & SILVER, H.K.B. (1989). Phase I-2 trial of Interleukin-2 (IL-2) splenic artery perfusion in advanced malignacy. *Proc. Am. Soc. Clin. Oncol.*, **8**, A686.

LISSONI, P., TANCINI, G., ROVELLI, F., CATTANEO, G., ARCHILI, C. & BARNI, S. (1990). Serum interleukin-2 levels in relation to the neuroendocrine status in cancer patients. *Br. J. Cancer*, **62**, 838–839.

MALKOVSKY, M., LOVELAND, B., NORTH, M. & 4 others (1987). Recombinant Interleukin-2 directly augments the cytotoxicity of human monocytes. *Nature*, **325**, 262–265.

MAVLIGIT, G.M., ZUKIWSKI, A.A., GUTTERMAN, J.J., SALEM, P., CHARNSANGAVEI, C. & WALLACE, S. (1990). Splenic versus hepatic artery infusion of Interleukin-2 in patients with liver metastases. *J. Clin. Oncol.*, **8**, 319–324.

MILLER, A.B., HOOGSTRATEN, B., STAQUET, M. & WINKLER, A. (1981). Reporting results of cancer treatment. *Cancer*, **47**, 207–214.

ROSENBERG, S.A., LOTZE, M.T., YANG, J.C., AEBERSHOLD, P.M., LINEHAN, W.M., SEIPP, C.A. & WHITE, D.E. (1989). Experience with the use of high-dose Interleukin-2 in the treatment of 652 cancer patients. *Ann. Surg.*, **210**, 474–485.

SELVAGGI, K.J., VLOCK, D.R., JOHNSON, J.T., SNYDERMAN, C.H., RUBIN, J., KIRKWOOD, J., HASELOW, R., LETESSIER, E., WHITESIDE, T. & PRESCOTT, K. (1990). Phase Ib trial of peritumoral and intranodal injections of IL-2 in patients with advanced squamous cell carcinoma of the head and neck – preliminary results. *Proc. Annul. Meet Am. Soc. Clin. Oncol.*, **9**, A691.

WALDMANN, W.A., GOLDMAN, C.K., ROBB, R.J., DEPPER, J.M., LEONARD, W.J., SHARROW, S.O., BONGIOVANNI, K.F., KORSMEYER, S.J. & GREENE, W.C. (1984). Expression of Interleukin-2 receptors on activated human B cells. *J. Exp. Med.*, **160**, 1450–1466.